# Manipulation of the crosstalk between tumor angiogenesis and immunosuppression in the tumor microenvironment: Insight into the combination therapy of anti-angiogenesis and immune checkpoint blockade

**DOI:** 10.3389/fimmu.2022.1035323

**Published:** 2022-11-10

**Authors:** Weiwei Zheng, Cheng Qian, Yu Tang, Chunmei Yang, Yueke Zhou, Peiliang Shen, Wenxing Chen, Suyun Yu, Zhonghong Wei, Aiyun Wang, Yin Lu, Yang Zhao

**Affiliations:** ^1^ Department of Biochemistry and Molecular Biology, School of Medicine & Holistic Integrative Medicine, Nanjing University of Chinese Medicine, Nanjing, China; ^2^ Jiangsu Key Laboratory for Pharmacology and Safety Evaluation of Chinese Materia Medica, School of Pharmacy, Nanjing University of Chinese Medicine, Nanjing, China; ^3^ Jiangsu Collaborative Innovation Center of Traditional Chinese Medicine (TCM) Prevention and Treatment of Tumor, Nanjing University of Chinese Medicine, Nanjing, China

**Keywords:** immune checkpoint blockade, anti-angiogenesis, tumor immune microenvironment, vessel normalization, targeted therapy

## Abstract

Immunotherapy has been recognized as an effective and important therapeutic modality for multiple types of cancer. Nevertheless, it has been increasing recognized that clinical benefits of immunotherapy are less than expected as evidenced by the fact that only a small population of cancer patients respond favorably to immunotherapy. The structurally and functionally abnormal tumor vasculature is a hallmark of most solid tumors and contributes to an immunosuppressive microenvironment, which poses a major challenge to immunotherapy. In turn, multiple immune cell subsets have profound consequences on promoting neovascularization. Vascular normalization, a promising anti-angiogenic strategy, can enhance vascular perfusion and promote the infiltration of immune effector cells into tumors *via* correcting aberrant tumor blood vessels, resulting in the potentiation of immunotherapy. More interestingly, immunotherapies are prone to boost the efficacy of various anti-angiogenic therapies and/or promote the morphological and functional alterations in tumor vasculature. Therefore, immune reprograming and vascular normalization appear to be reciprocally regulated. In this review, we mainly summarize how tumor vasculature propels an immunosuppressive phenotype and how innate and adaptive immune cells modulate angiogenesis during tumor progression. We further highlight recent advances of anti-angiogenic immunotherapies in preclinical and clinical settings to solidify the concept that targeting both tumor blood vessels and immune suppressive cells provides an efficacious approach for the treatment of cancer.

## 1 Introduction

The last decade has undoubtedly visualized the striking rise of immunotherapy, in particular immune checkpoint blockade (ICB) therapy, for the treatment of cancer. In contrast to conventional cytotoxic agents, which directly target cancer cells, a major goal of cancer immunotherapy is to alleviate tumor-associated suppression of anticancer immune responses. A significant portion of cancer immunotherapy research has focused on heightening the functions of effector T cells, which play a direct role in recognizing tumor-associated antigens and in mediating tumoricidal responses (1, 2). The immune checkpoint mediators, such as cytotoxic T lymphocyte-associated protein 4 (CTLA-4), programmed death 1 (PD-1) as well as programmed death-ligand 1 (PD-L1) have been validated to be effectively targeted and their antibodies have been approved by the Food and Drug Administration (FDA) for treating various types of cancer. To date, the development of ICB-based immunotherapy has remarkably transformed the current therapeutic paradigm in oncology (3). However, ICB therapy commonly benefits <15% of cancer patients and leads to immune-associated adverse effects (4, 5). An alternative immunotherapy, utilizing engineered chimeric antigen receptor (CAR) T-cells to specifically target tumor-associated antigens, has opened the door to a promising novel treatment of numerous “liquid” cancers and achieved similar success in solid tumors (6). Among numerous crucial factors of immunity to cancer, the tumor microenvironment (TME) serves as a predominant challenge that overtly diminishes the effectiveness of ICB. The interactions between tumor endothelial cells (ECs) and immunosuppressive immune cells tend to form a vicious cycle that extensively distorts anti-tumor immune response and aggravates the development of tumors in the TME (7, 8). Notably, tumor blood vessels are prone to facilitate the infiltration of immunosuppressive immune cells into tumors, which in turn fuels tumor angiogenesis (9). This deteriorative crosstalk between immune suppression and angiogenesis not only produces the endothelium that brakes the penetration of T cells into tumors, but also curtails the functions of T cells and even results in elevated apoptosis of T cells. To this end, manipulation of tumor blood vessels is inclined to act as a reliable strategy to boost anti-tumor immune response and counteract the resistance to ICB (10).

In comparison to ICB, anti-angiogenesis therapy gained substantial attention at an earlier stage (11). It has been well established that neovascularization or angiogenesis plays a pivotal role in maintaining homeostasis due to the fact that blood vessels are able to transport nutrients to body’s tissues and organs and remove metabolic wastes (12). Of note, excessive growth of blood vessels tends to aggravate the progression of a variety of diseases especially tumors and intraocular vascular diseases (13). In light of therapy, conventional anti-angiogenic strategy aims to destroy tumor blood vessels (14). Nevertheless, the therapeutic outcomes for cancer patients received anti-angiogenesis therapy alone in clinic are less than what is expected. Excessive suppression of blood vessel formation in tumors confers diminished vascular perfusion, which sets up difficult hurdles for immune cell infiltration and drug delivery (9, 15). Vessel normalization theory, firstly proposed by Rakesh Jain, offers a novel and promising perspective in terms of anti-angiogenesis and shows potential synergistic effect in combination with other therapies (16). More intriguingly, vascular normalization and immune reprogramming can generate a positive feedback, which implies that reinforcement in one side has high propensity to strengthen the other’s effects (9).

In this review, we mainly discuss the latest advances on how abnormal tumor vasculature modulates the infiltration of various types of immune suppressive cells and incites an immunosuppressive phenotype, as well as outline how immunosuppressive TME influences tumor angiogenesis. We further gain insight into the latest knowledge of ICB in combination with anti-angiogenesis therapy and highlight the advances of relevant clinical trials.

## 2 Manipulation of angiogenesis has pivotal impacts on immunosuppressive tumor microenvironment

### 2.1 Description of blood vessel formation in tumors

It has been widely held that the hypoxic microenvironment in solid tumors results in rapid but abnormal blood vessel establishment. In fact, tumor angiogenesis is regarded as an extremely complicated process (17). This is owing to (a) the perturbed equilibrium between pro-angiogenic and anti-angiogenic factors, leading to the so-called “angiogenic switch” with excessive pro-angiogenic signaling (**Table 1**). For instance, vascular endothelial growth factor (VEGF) as the major factor to initiate angiogenesis is substantially up-regulated upon the stimulation of hypoxia. It plays a vital role in strengthening the formation of blood vessels mainly through leading to the activation of VEGF receptor-2 (VEGFR-2) that is predominantly expressed by ECs. The activation of VEGFR-2 is inclined to trigger the transduction of multiple critical signaling pathways, resulting in specific endothelial responses such as cell survival, proliferation, migration as well as vascular permeability (13). Similarly, fibroblast growth factor (FGF) and its receptor (FGFR) exert significant effects on propelling the proliferation, migration, and survival of ECs, thereby contributing to elevated angiogenesis (47). (b) angiogenic signaling cascades are usually located at the downstream of oncogene activation. Accordingly, tumor blood vessels are rather blind ended as well as leaky with disrupted endothelial junctions and disorganized endothelial lining, and display aberrant basement membrane and poor pericyte coverage (48). These characteristics seem eventually to be hallmarks of immature and dysfunctional tumor vasculature, inciting the consequence that tumor parenchyma maintains constantly hypoxic, which renders a negative feedback loop whereby pro-angiogenic signals never cease (49). Dysfunctional tumor blood vessels featured with reduced vascular perfusion end up producing swollen and thick vessel wall, where clotting events and hemostasis frequently occur. Thus, tumors with intensified vascular density are prone to be highly hypoxic and vice versa, depending on their vascular functionality and metabolic demand (50).

**Table 2:** The main angiogenic modulators involved in angiogenesis.

Angiogenesis regulators	Signaling pathways	Functions	Anti-angiogenic agents	Refs
VEGF	Activate RAF/MEK/MAPK and PI3K/AKT signaling pathways	Proliferation, migration and invasion	Bevacizumab/Ramuciruma/Pazopanib/Sunitinib Axitinib/Zivaflibercep/Lenv-atinib	(13, 18–20)
	Deactivate NFAT/β-catenin/VE-cadherin and eNOS	Increase vascular permeability		
	Upregulate the expression of epithelial mesenchymal transition-related genes	Promote vasculogenic mimicry		
FGF	Activate RAS/RAF/MAPKK/MAPK signaling pathway	Proliferation	FP-1039/NSC12/SSR128129E/AZD4547/BGJ398/LY287445/FPA144	(21–24)
	Activate PI3K/AKT/FOXO signaling pathway	Survival		
	Activate IP3/NFAT signaling pathway	Stimulate cell motility		
PDGF	Activate JAK/STAT, PI3K, PLC-γ and MAPK signaling pathways	Proliferation, migration and extracellular matrix synthesis	SU6668/Imatinib/Lartruvo/Olaratumab	(25, 26)
ANG2	Inhibit PI3K/AKT/FOXO1 signaling pathway	Increase vascular permeability	Trebananib/Nesvacumab/MEDI3617/AMG780/AKB-9778	(27–30)
HGF	Activate PI3K, PLC-γ, Crk/CRKL, Ras/Raf/MEK/ERK and Rac1 signaling pathways	Proliferation, adhesion, motility, division and survival	Ficlatuzumab/AMG337 BMS-777607/Cabozantinib/Crizotinib/Foretinib/LY2801653/LY2875358/SAIT301/SAR125844/Rilotumumab/Onartuzumab	(31–33)
TGF-β	Activate Smad2/Smad3 signaling pathway	Promote tumor angiogenesis	Luspatercept	(34, 35)
IFN-α	Inhibit SP1 and SP3 expression levels	Reduce angiogenesis	–	(36, 37)
	Activate PI3K/AKT/mTOR signaling pathway	Promote vasculogenic mimicry		
TNF-α	Activate PI3K, p38, JNK, ERK and NF-κB signaling pathways Activate PIGF/VEGFR1 and VEGFA/VEGFR2	Promote tumor angiogenesis	Lenalidomide/Thalidomide	(38)
IL-1	Activate MAPK and IKK/NF-κB signaling pathways	Promote tumor angiogenesis	–	(39)
IL-1β	Activate JNK and p38 MAPK signaling pathways	Promote tumor angiogenesis	–	(40)
IL-33	Activate ST2/TRAF6-Akt-eNOS signaling pathway phosphorylate VE-cadherin	Increase vascular permeability and promote angiogenesis	–	(41)
IL-18	Activate Src and JNK signaling pathways	Promote angiogenesis	–	(42)
IL-6	Activate IL-6/STAT3/VEGFA signaling pathway	Promote angiogenesis	Tocilizumab/Sarilumab/Siltuximab	(43, 44)
IL-8	Activate PI3K/Rac1/RhoA signaling pathway	Promote angiogenesis	–	(45, 46)

VEGF vascular endothelial growth factor, FGF fibroblast growth factor, PDGF platelet-derived growth factor, ANG2 angiopoietins2, HGF hepatocyte growth factor, TGF-β transforming growth factor-beta, IFN-α interferon-α, TNF-α tumor necrosis factor-α, IL-1 interleukin-1, IL-1β interleukin-1β, IL-33 interleukin-33, IL-18 interleukin-18, IL-6 interleukin-6, IL-8 interleukin-8.

**Figure 1:**
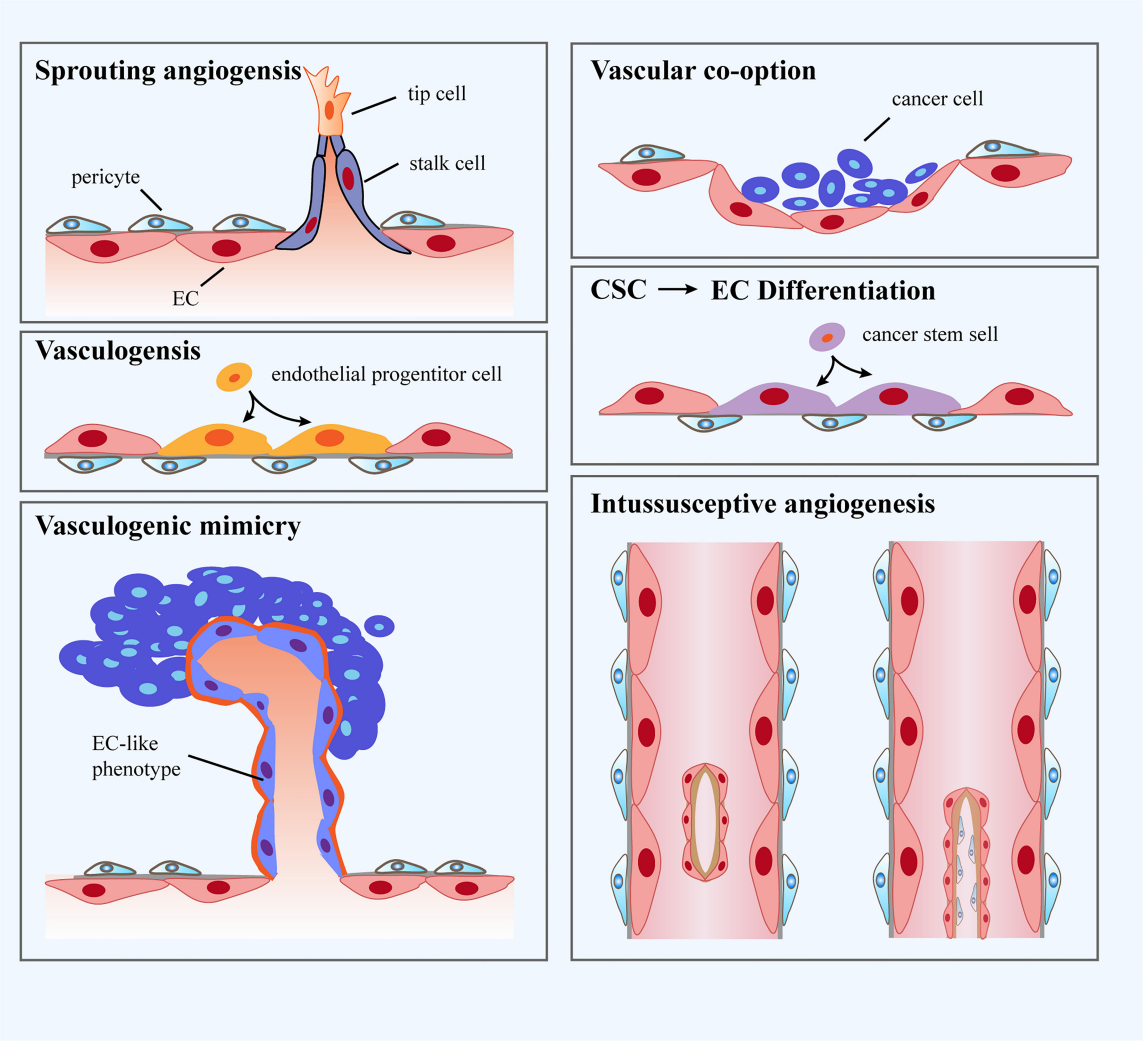
Classification of blood vessel formation. i: Sprouting angiogenesis refers to a process involving production and outgrowth of sprouts (tip cells), which ultimately integrate with an existing vessel or newly formed sprout. ii: Vasculogenesis is prenatal neo-vascularization occurred from endothelial progenitor cells (EPCs). The EPCs proliferate and generate lumens, ultimately gathering into new blood vessels. iii: Vasculogenic mimicry is regarded as a matrix-embedded blood vessel network produced by tumor cells rather than endothelial cells (ECs). iv: Vascular co-option represents that tumors grow by hijacking preexisting vessels in the peritumoral tissues. v: Trans-differentiation of cancer stem cells (CSCs) reflects neo-vascularization in tumors through differentiating CSCs to ECs. vi: Intussusceptive angiogenesis refers to the formation of new vasculature where a pre-existing vessel splits into two.

It has been well accepted that the formation of tumor blood vessels is able to occur in an array of manners, including sprouting angiogenesis, vasculogenesis, vessel co-option, vasculogenic mimicry, intussusceptive angiogenesis and trans-differentiation of cancer cells (**Figure 1**). In fact, angiogenesis and vasculogenesis are the predominant modes of tumor angiogenesis. Angiogenesis, the most widely investigated pattern of new vessel formation in tumors, refers to the initiation of tumor blood vessels from existing ECs and the production of new neoplastic capillaries in the format of sprouting (51). Indeed, sprouting angiogenesis is triggered by a panel of pro-angiogenic growth factors (eg. VEGF) produced by hypoxic and nutrient-deprived microenvironment, which allow quiescent ECs to exhibit activated phenotype. Further, activated ECs tend to release matrix metalloproteinases (MMPs) to degrade the basement membrane and turn to invasive profile (52), which enables tip cells to protrude and migrate towards the core of the angiogenic stimulus. Tip cells with minimal proliferative capability extend filopodia and lamellipodia to lead the nascent sprout towards oxygen-deprived regions. In addition, a wealth of molecules associated with extracellular matrix (ECM) degradation and basement membrane deposition have been observed to be highly expressed in tip cells (53). Tip cells are followed by another type of ECs named stalk cells, which proliferate to propel the elongation of sprouts and strengthen the formation of lumens. Specification in migratory tip versus proliferating stalk cell is overtly dynamic, resulting in that ECs persistently compete for the lead position. Upon the condition that two tip cells from adjacent sprouts meet, they are inclined to anastomose to generate a perfused new vessel (54). Vasculogenesis is achieved *via* the recruitment of endothelial progenitor cells (EPCs) that are capable of differentiating into ECs and penetrating into tumors to be directly involved in the generation of tumor blood vessels (55). Vasculogenic mimicry is a new tumor microcirculation mode that is different from the classical patterns of tumor angiogenesis as it relies on tumor cells rather than ECs (56). Intussusceptive angiogenesis is frequently observed in the lumen of existing blood vessels and is governed by the interstitial columnar structure, leading to the incision of the original vascular lumen and the production of new blood vessels. More specifically, it splits pre-existing vessels to induce new blood vessel formation (57). Vessel co-option has preference to appear in various malignancies, which means that tumor cells migrate along the existing or newly triggered blood vessels (hijacking the vasculature) to support tumor growth and metastasis (58). Vessel co-option can be visualized in a plethora of types of tumors in humans at multiple anatomic sites and influences the prognosis of cancer patients, but it still remains a frequently overlooked phenomenon (59). Trans-differentiation of cancer cells is recognized as the trans-differentiation of cancer stem cells (CSCs) to ECs and vascular smooth muscle-like cells, yielding the occurrence of neovascularization (60). Furthermore, intussusceptive angiogenesis is recognized as a faster phenomenon to orchestrate a plexus compared to sprouting angiogenesis. Intussusceptive angiogenesis has been deemed to be less energetically demanding, which allows it to minimally rely on the migration and proliferation of ECs (61).

### 2.2 Tumor angiogenesis creates immunosuppressive microenvironment

**Figure 2:**
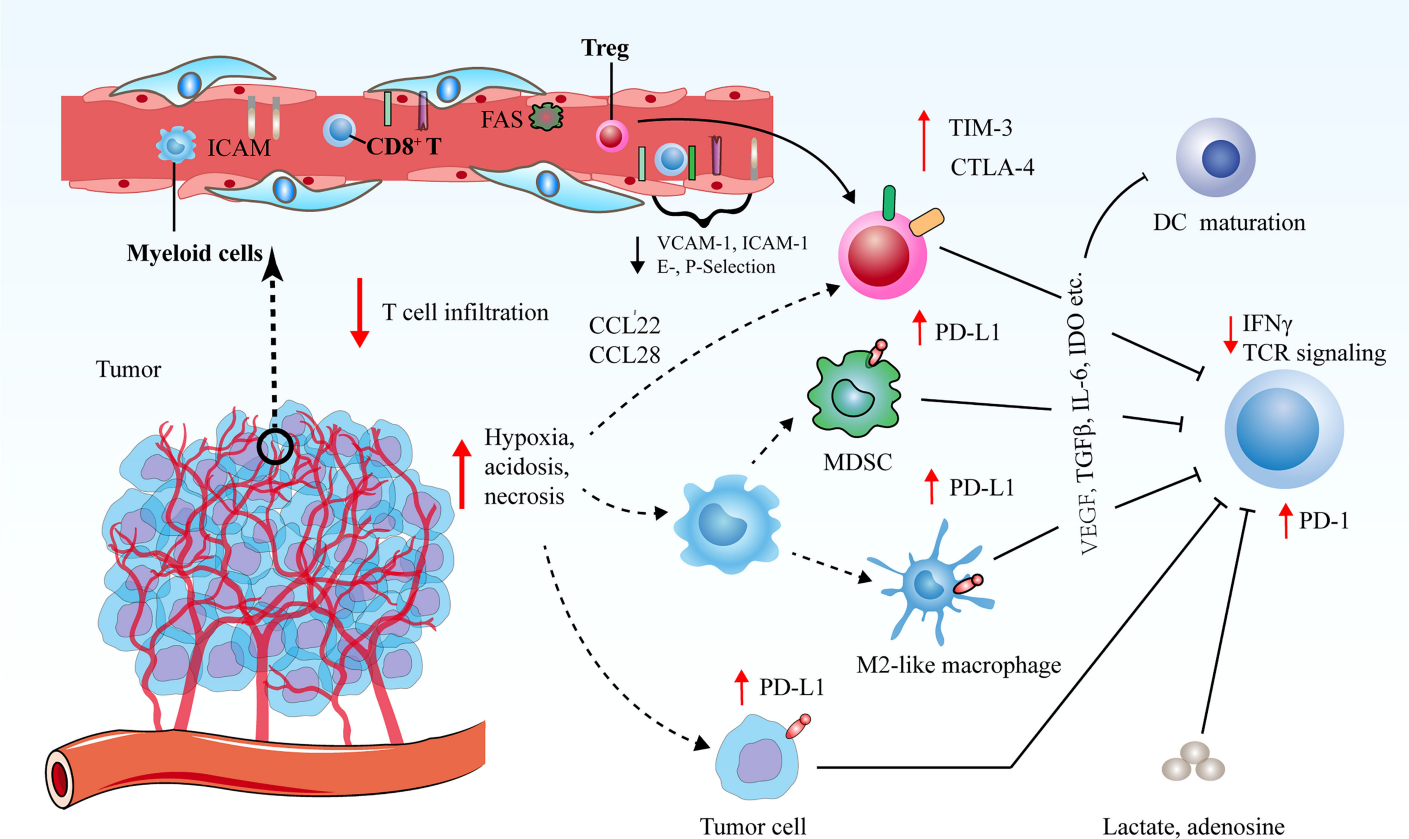
Tumor angiogenesis confers immunosuppressive tumor microenvironment *via* various routes. Decreased vascular perfusion and elevated vascular permeability augment tumor hypoxia, acidosis and necrosis, which initiate various immune suppressive processes to mitigate effector T cell functions. Hypoxia not only induces the secretion of cytokines and chemoattractants to promote the recruitment of immunosuppressive cells, but also boost the expression levels of CTLA-4 or TIM-3 on Tregs, and PD-L1 on MDSCs, TAMs and tumor cells to restrict the function of immune-supportive cells. The ECs of tumor vessels with lower levels of cell adhesion molecules are capable of triggering endothelial anergy, thereby inhibiting the infiltration of effector T cells into tumors.

In the majority of solid tumors, uncontrolled angiogenesis generates immunosuppressive microenvironment *via* influencing an array of immune-associated steps (**Figure 2**) (62–64). On one hand, hyperactive and aberrant angiogenesis impedes the number and tumor-killing activity of tumor-infiltrating lymphocytes (TILs). Firstly, leaky and dysfunctional blood vessels with poor pericyte coverage and abnormal basement membrane support underlines high interstitial fluid pressure (IFP), which indicates that there is elevated pressure difference to overcome for T cell trafficking into tumors. This undoubtedly leads to that simply fewer T cells are able to transmigrate across the physical barrier and infiltrate into tumor bed. Secondly, a bunch of critical adhesion molecules including vascular cell adhesion molecule 1 (VCAM-1) and intercellular adhesion molecule 1 (ICAM-1) expressed on ECs are significantly down-regulated in newly formed tumor blood vessels, which hampers the adhesion and transmigration of T cells (65). Thirdly, neovascularization fails to compensate for enhanced oxygen consumption, and concurrent hypoxia potently declines the functions of TILs as a result of that hypoxia increases the expression of multiple inhibitory mediators for anti-tumor immune response, such as PD-L1, indoleamine 2, 3-dioxygenase (IDO), interleukin-6 (IL-6), and interleukin-10 (IL-10) (11). Hypoxia is also inclined to trigger the expression levels of immune checkpoint molecules including PD-L1 on tumor cells as well as PD-L1, T cell immunoglobulin and mucin-domain containing-3 (TIM-3) and CTLA-4 on tumor-associated macrophages (TAMs), myeloid-derived suppressor cells (MDSCs) and regulatory T cells (Tregs) (66, 67). Moreover, upon the stimulation of VEGF, hypoxia tends to indirectly upregulate the expression of PD-1 on CD8^+^ T cells, thus further inhibiting immune effector cell activation and function Moreover, hypoxia together with tumor cell necrosis gives rise to the elevated the extracellular concentrations of the immune-suppressive metabolites such as adenosine and lactate. The enhanced level of lactate further results in metabolic lactic acidosis (a low pH in the blood due to accumulation of lactic acid) and leads to declined CD8^+^ T cell function by interfering with T cell receptor (TCR)-induced interferon-γ (IFN-γ) production (68, 69). Lastly, the high expression of Fas ligand (FasL) on tumor ECs selectively eliminates effector CD8^+^ T cells instead of Tregs, which is attributed to the augmented level of cellular FLICE-inhibitory protein (c-FLIP) on Tregs (70).

On the other hand, excessive angiogenesis tends to promote the abundance of pro-tumor immune subsets that are crucial for immunosuppressive microenvironment. Upon expansion of abnormal tumor blood vessels, hypoxic microenvironment in tumors leads to the upregulation of chemokine (C-C motif) ligand-22 (CCL-22) and chemokine (C-C motif) ligand-28 (CCL-28), which are both involved in the recruitment of Tregs into tumors (71, 72). Additionally, hypoxia promotes the accumulation of MDSCs and facilitates the differentiation and polarization of TAMs into an immunosuppressive M2-like phenotype (73). In addition, circulating VEGF hinders the maturation and function of dendritic cells (DCs) to enable tumor cells to escape immune surveillance (74). These processes collaborate to hamper the maturation of effector immune cells and propel the anergy and exhaustion of T cells. Therefore, dysfunctional tumor vessels play an indispensable role in yielding the immunosuppressive nature of the TME and limiting the effects of cancer immunotherapies.

## 3 Different types of immune cells exert versatile effects on modulating tumor angiogenesis

### 3.1 Innate immune cells regulate tumor angiogenesis

It has been widely held that tumors, partially due to their hypoxic and acidic property, are able to recruit a large number of distinct innate immune cells that constitute approximately 30% of the total cell populations in tumors (75). Notably, tumor-associated neutrophils (TANs), TAMs, MDSCs, DCs and mast cells appear to be the most frequently observed cell populations and are correlated to intra-tumoral vascular density (8, 76–82). Indeed, in addition to cancer cells and cancer-associated fibroblasts, myeloid cells have been perceived as a crucial source of growth factors and chemokines to influence tumor vessels and determine the endothelial phenotype and function (83, 84), which has been substantiated in various mouse tumor models, such as breast, skin, brain, and cervical tumors (76). Additionally, certain classes of innate immune cells are capable of interfering with and polarizing other specific groups of immune cells to obtain either pro-angiogenic or anti-angiogenic properties, further influencing the amount and grade of angiogenesis in tumors (85). An increasing number of striking studies using murine tumor models have demonstrated the functional significance of innate immune cells in modulating tumor angiogenesis and subsequent tumor growth and metastasis, some of which are highlighted in detail as follows.

#### 3.1.1 TANs

It has been well known that neutrophils are prone to give rise to the production of either pro-angiogenic or anti-angiogenic factors (86). Despite that neutrophils are featured with an eventually differentiated phenotype with short half-life property, these cells are still able to harbor a certain type of plasticity that enables them to differentiate into two different subpopulations: type 1 neutrophils (N1) with anti-microbial function, and TANs or N2 neutrophils with conspicuous pro-tumor and pro-angiogenic characteristics in the presence of TGF-β (87). It has been widely recognized that N1 neutrophils exert central effects on the front-line defense against harmful invading pathogens. They are able to either phagocytose micro-organisms, secrete soluble anti-microbial molecules from the granules or propel the formation of neutrophil extracellular traps (NETs), which are classic web-like fibers that consist of chromatin and serine proteases, playing a crucial role in trapping and destructing the extracellular microbes (88, 89). It has been reported that TANs/N2 neutrophils are able to be converted into N1 neutrophils in both mouse lung cancers and human melanomas following the stimulation of interferon-β (IFN-β) (90). In the context of malignant tumors, TANs tend to predominantly produce a list of pro-angiogenic factors including VEGF, FGF and MMPs as well as generate proteases (86, 91–93). Interestingly, neutrophils are known to contain VEGF-enriched granules that are released following the stimulation of TNF and create a reliable pathway of VEGF accessibility to improve the expansion of blood vessels (94). In the tumor microenvironment, TANs were also demonstrated to generate matrix metalloproteinase-9 (MMP-9) to orchestrate sequestered VEGF from the ECM in dysplastic pancreatic islet lesions of Rip1Tag2 transgenic mouse model. The produced VEGF initiated the onset of angiogenesis in these premalignant lesions and allowed the malignant transformation and progression (95–97). In line with this observation, the depletion of TANs with antibodies against Gr1 dramatically repressed the angiogenic switch in the pancreatic islet lesions (96). Moreover, neutrophils stimulated with colony-stimulating factor (G-CSF) promoted the expression levels of the angiogenic factor Bv8 (prokineticin-2) in various tumor models that boosted the proliferation, migration and survival of ECs, thereby generating a positive feedback loop for strengthening the recruitment and activation TANs (98).

#### 3.1.2 TAMs

Macrophages are deemed to work as the professional phagocytes of innate immune cells with distinct specific roles, relying on the type of danger signals and endogenous molecules to which they are exposed. Macrophages are capable of triggering inflammatory responses and collaborating with other immune cells to activate adaptive T lymphocyte responses through antigen processing and presentation, thereby acting as the sentinels in all tissues of the body against invading pathogens. In tumors, it has been revealed that TAMs preferentially stay pro-tumorigenesis M2/T helper (Th) 2-like status, however, polarization of macrophages to a pro-inflammatory and anti-tumorigenesis (M1/Th1-like) phenotype tends to lead to impaired angiogenesis in multiple mouse and human tumor models (99–101).

Indeed, it was uncovered that M1-phenotypic macrophages mitigated sprouting angiogenesis and yielded the maturation of blood vessels through inducing the secretion of numerous anti-angiogenic cytokines including interleukin-12 (IL-12) and TNF-α (102, 103). Interestingly, IL-12 produced from M1 form of TAMs was capable of skewing TAMs to the M1 phenotype, therefore generating a positive feedback loop aiming at driving the anti-angiogenic M1 pattern. To this end, IL-12 administration not only curtailed microvascular density but also polarized TAMs from M2 towards M1 phenotype in tumors (102, 104). Moreover, M2-phenotypic TAMs are inclined to strengthen tumor angiogenesis *via* potentiating the secretion of multiple pro-angiogenic growth factors (e.g. VEGF, FGF, EGF, and PDGF-b), angiogenic chemokine (C-X-C motif) ligands (e.g. CXCL-8 and CXCL-12), as well as angiogenesis-associated elements (e.g. TGF-β, TNF-α, and thymidine phosphorylase) (105). These imperative factors not only have preference to reinforce EC proliferation and migration but also polarize TAMs away from tumor-suppressing M1 manner to tumor-promoting M2 manner.

Qiu and colleagues demonstrated that M2-like TAMs were positively correlated with the microvascular density in human pancreatic cancer. More specifically, the exosomes derived from M2-like TAMs were able to deliver miR-155-5p and miR-221-5p into ECs, which boosted the angiogenesis in pancreatic ductal adenocarcinoma (PDAC) through targeting E2F2 (106). Chen and co-workers highlighted that M2 TAMs-derived exosomal miR-942 tended to escalate angiogenesis *via* binding to FOXO1 to alleviate the suppression of β-catenin as well as harness the migration and invasion of lung adenocarcinoma (LUAD) cells (107). Pollard et al. also unveiled that M2-phenotypic TAMs had high propensity to propel the progression of tumor angiogenesis, they found that the infiltration of TAMs into the parenchyma of primary tumors was potently enhanced shortly prior to the development of a dense vascular network in the tumor area. As such, blockade of the infiltration of macrophages into tumors repressed the angiogenic switch (97). It has been well accepted that M2-phenotypic TAMs are the more predominant subset compared with M1-phenotypic TAMs in most solid tumors. In this regard, inactivation of TAMs *via* counteracting the CSF1/CSF1R signaling pathway, extensive antagonization of TAMs through clodronate liposomes, or genetic depletion of VEGF in TAMs was able to impede the development of angiogenic switch. In contrast, restoration of the macrophages through genetic approaches was capable of rescuing the angiogenic phenotype in tumors (97, 108–111).

#### 3.1.3 MDSCs

As defined by the morphology and function, immature myeloid cells that can be classified into two main subtypes: granular MDSC (G-MDSC) and monocytic MDSC (M-MDSC). The studies focusing on the role of MDSCs in tumors showed that the main capabilities of these cells are to inhibit immunity by perturbing both innate and adaptive immune responses as well as to strengthen angiogenesis (76, 112). MDSCs have been gaining growing attention in curbing immune response in tumors owing to their ability to suppress the infiltration and activity of T cells (113, 114). In light of angiogenesis, MDSC-released VEGF was sufficient to prohibit the expression levels of various key adhesion molecules including ICAM-1 and VCAM-1 in tumor-associated ECs, thereby preventing the adhesion and extravasation of T cells (115, 116). MDSCs promote angiogenesis through heightening the levels of IL-10 but diminishing the leves of IL-12 in the TME. Furthermore, MDSCs are capable of enhancing angiogenesis by giving rise to the productions of Bv8 and MMP-9. MDSC-derived Bv8 is able to directly fuel the formation of neovessels through endocrine gland-derived VEGF1 (EG-VEGF1) and VEGF2 (EG-VEGF2) and can also further accumulate MDSCs within the tumors. Of note, a series of important inflammatory factors including granulocyte G-CSF, IL-1β and IL-6 are prone to confer the recruitment, activation and expansion of MDSCs in tumors through inducing the activation of STAT3 signaling pathway, thus further driving the pro-angiogenic property of MDSCs while hindering their differentiation potential into neutrophils or macrophages. Hence, it seems to imply that it is not surprising that the pro-angiogenic expression characteristics and functions of MDSCs remarkably overlap with those of mature macrophages (92, 117, 118).

#### 3.1.4 DCs

DCs as another crucial innate immune subpopulation in the TME have also been revealed to play a pivotal role in modulating tumor angiogenesis on the basis of their maturation status (119). It has been increasingly recognized that DCs are prone to prime naiüve T cells and further lead to the activation of antigen-specific immunity. In fact, human DCs contain two types of predominant subsets, which are myeloid dendritic cells (MDCs) and plasmacytoid dendritic cells (PDCs). MDCs expressing tumor antigens have been employed in clinical trials to confer prominent therapeutic responses against various types of tumors. Zou et al. demonstrated that MDCs were observed to be absent from malignant ascites. Further, they showed that MDCs exerted striking effect on hampering tumor angiogenesis *via* promoting the secretion of IL-12 (120). On the contrary, a large number of PDCs and high level of stromal-derived factor including CXCL-12/SDF-1 were found to be present in their malignant ascites, further enabling PDCs to penetrating into the TME. It was shown that tumor-associated PDCs triggered tumor angiogenesis *via* provoking the secretion of TNF-α and IL-8 (120). Notably, the most predominant subpopulation of DCs in the TME is deemed to be immature DCs (iDCs) as cancer cells are able to preferentially recruit iDCs from peripheral blood vessels by producing a wealth of cytokines (e.g. VEGF, β-defensin, CXCL-12, HGF, and CXCL-8) (121). Moreover, iDCs are endowed with pro-angiogenic property and exert indispensable effect on bolstering tumor angiogenesis *via* secreting multiple angiogenic factors including VEGF-A and FGF (122).

#### 3.1.5 Mast cells

MCs, a population of multifunctional immune cells that were originally identified in human tumors by Paul Ehrlich in the 1870s, are derived from bone marrow and are also thought to be involved in tumor angiogenesis (123). Indeed, there are considerable mast cells that are observed in tumors and peritumor tissues of cancer patients, and their functions in cancer insurgence and progression are dependent on tumor types. Intriguingly, mast cells releases a set of pro-angiogenic factors including FGF-2, VEGF-A, TNFs, CXCL-8 (123) and various proteases including MMPs (predominantly MMP-9), as well as chymase and tryptase that enable pro-MMPs to become their active forms (124, 125). Multiple lines of evidence highlighted that mast cell proteases serve as the pivotal regulators in rendering tumor angiogenesis (125). Furthermore, Li et al. showed that recruitment of mast cells into tumor parenchyma contributed to elevated angiogenesis in both *in vitro* and *in vivo* models. Mechanistic studies deciphered that infiltrated mast cells were validated to orchestrating tumor angiogenesis by activating PI3K/AKT/GSK3β/AM signaling cascade in the renal cell carcinoma (126).

### 3.2 Adaptive immune cells influence tumor angiogenesis

In fact, the function of lymphocytes in regulating tumor angiogenesis are still ambiguous. Nevertheless, a growing number of studies have documented that lymphocytes are able to govern the growth and maturation of tumor blood vessels in a direct or indirect manner.

#### 3.2.1 B lymphocytes

Although the role of B lymphocytes in curbing tumor angiogenesis and the underlying mechanisms are still obscure, an increasing number of studies have gradually unveiled that the functions of B lymphocytes in tumor angiogenesis vary under distinct situations. For example, Yang and colleagues elucidated that tumor-associated B lymphocytes with activated STAT3 resulted in the accelerated tumor progression by virtue of augmenting tumor angiogenesis (127). Additionally, the amount of B cells in tumor tissues was positively correlated with the expression levels of a list of pro-angiogenic molecules that are located at the downstream of STAT3, as well as the extent of tumor angiogenesis. Indeed, it has also been documented that B lymphocytes are capable of potently strengthening tumor angiogenesis *via* promoting the secretion of an array of critical pro-angiogenic factors including VEGF, FGF-2, and MMP-9 (127), or indirectly *via* skewing the polarization of TAMs towards an pro-angiogenic and immunosuppressive M2-phenotypic TAMs in an IgG-dependent manner (128). Furthermore, Christofori and co-workers observed that the levels of a type of bone marrow-derived cell population, CD45^dim^VEGFR1^-^CD31^low^, were significantly elevated in the peripheral blood from multiple mouse models of tumor angiogenesis compared with that from healthy mice. More interestingly, gene expression profile analysis identified CD45^dim^VEGFR1^-^CD31^low^ cells as an immature B lymphocyte population. Long-term treatment with potent compounds (BIBF-1120, nintedanib) that inhibiting various pro-angiogenic factors (VEGF, FGF and PDGF) resulted in a striking impairment in the number of immature B lymphocytes in the blood from tumor-bearing mice as well as prominent reductions in tumor volume and microvascular density. Hence, CD45^dim^VEGFR1^-^CD31^low^ cell population may emerge as surrogate marker for tumor angiogenesis and be able to reflect the therapeutic response to anti-angiogenic strategies (129).

#### 3.2.2 T lymphocytes

**Figure 3:**
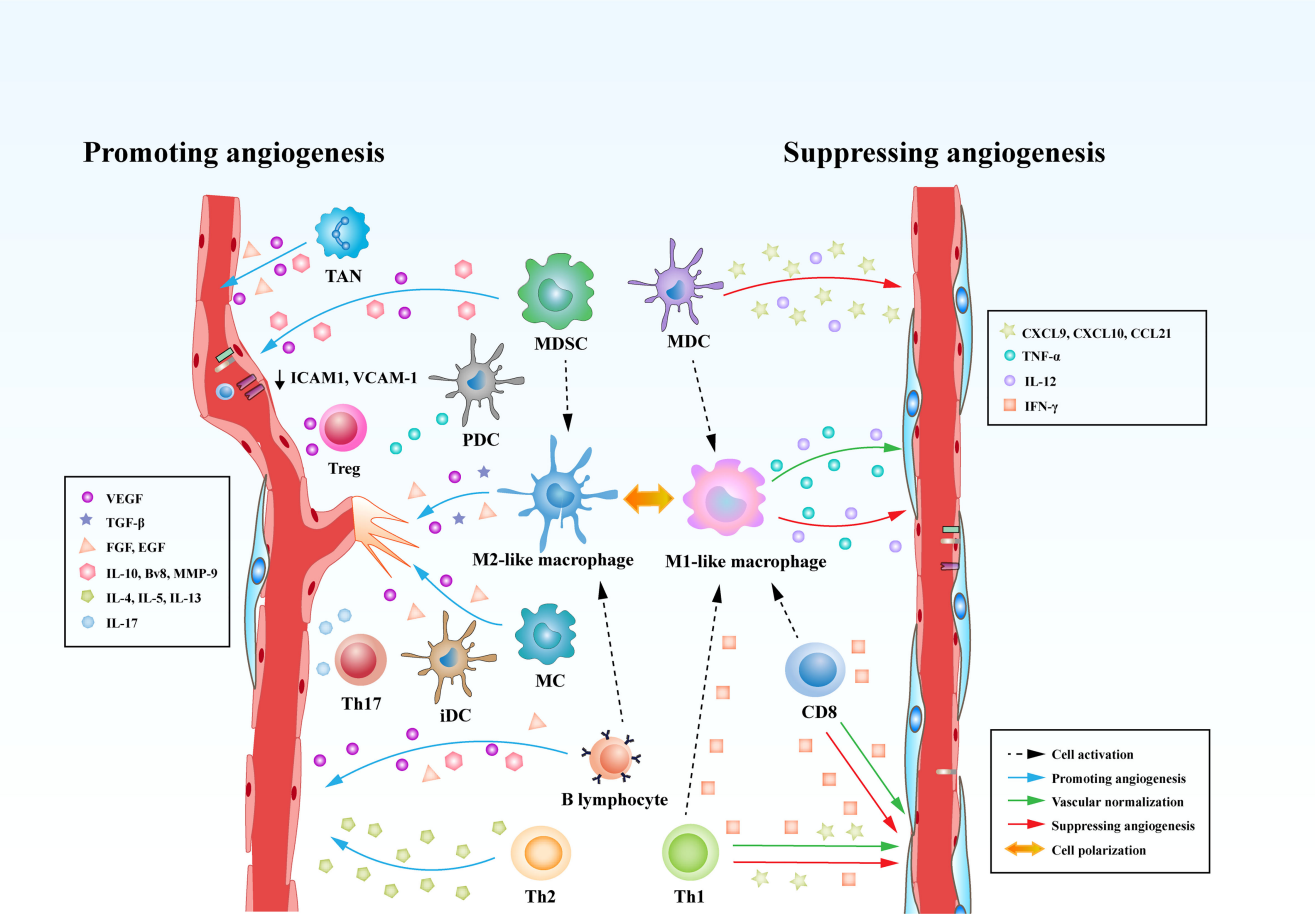
Immune cells play versatile roles in regulating tumor angiogenesis. Immune cells are able to directly curb the phenotypes and functions of tumor blood vessels through modulating the production of various cytokines. Some types of innate immune cells, such as MDCs and M1-like macrophages, produce cytokines (IL-12 or TNF) and chemokines (CXCL-9, CXCL-10, or CCL-21) that suppress tumor angiogenesis. Meanwhile, adaptive immune cells, such as CD8^+^ T cells and T helper 1 (Th1) cells, are capable of secreting IFN-γ, which is a potent cytokine that inhibits angiogenesis and induces vascular normalization in the TME. However, MDSCs, M2-like TAMs robustly augment tumor angiogenesis by secreting multiple factors including VEGF, IL-10, Bv8, and MMP-9. Moreover, Tregs, Th2, and Th17 cells can release pro-angiogenic factors such as VEGF, IL-4, IL-5, IL-13, and IL-17 to initiate the angiogenic switch. In addition to the direct effects on tumor vasculature, immune cells can also influence tumor vasculature indirectly through communicating and interfering with each other. CD8^+^ T cells and Th1 cells are able to skew macrophage polarization away from the M2 to the M1 phenotype. However, MDSCs and B lymphocytes reprogram TAMs from M1 to M2-like profile.

In comparison to B lymphocytes, T lymphocytes are prone to positively or negatively control tumor angiogenesis on the basis of the types of T cells. It has been shown that CD8^+^ T cells and CD4^+^ Th1 cells yield IFN-*γ* that limits the proliferation of ECs and triggers the generation of the angiostatic chemokines including CXCL-9, CXCL-10 and CXCL-11 in TAMs (91, 130). Another preponderant role of IFN-γ in influencing tumor angiogenesis is the polarization of TAMs from M2- to M1-phenotypic TAMs. It has been shown that hyperactive IFN-γ/STAT1 signaling cascade propels the reprograming of TAMs to M1-like profile, contributing to vascular remodeling and subsequent tumor destruction (83, 131). In contrast, Th2 cells as one type of CD4^+^ T cells play an important role in provoking profound tumor angiogenesis. Th2 cells expressing IL-4, IL-5, and IL-13 tend to promote the recruitment of M2-like TAMs *via* activating STAT6 and eventually instruct tumor angiogenesis (130). Th17 cells, another subtype of CD4^+^ T cells, have been demonstrated to be related to intensify angiogenesis in various types of human cancer. The expression of IL-17 in Th17 cells are associated with the infiltration of ECs and the formation of abnormal tumor blood vessels (132, 133). Tregs exert significant effects on inhibiting the function of IFN-*γ*-expressing CD4^+^ Th1 cells and producing the secretion of VEGF through hypoxia-mediated CCL-28, which both strikingly result in a pro-angiogenic tumor microenvironment (72) (**Figure 3**).

## 4 Intrinsic interactions between vascular normalization and immunotherapy

### 4.1 Advancements of anti-angiogenic strategies: From tumor starvation to vascular normalization

Systemic anti-angiogenic therapies have been well established with the rationale that suppressing the formation of tumor blood vessels is prone to confer conspicuous vascular eradication, consequently starving tumors to death due to disruption of delivery route for oxygen and nutrients or driving them to be “dormant”. In 1993, Napoleone Ferrara’s group uncovered a remarkable decrease in microvascular density and dramatic retardation of tumor growth in the nude mice harboring xenografts of glioblastoma multiforme, rhabdomyosarcoma and leiomyosarcoma following the treatment of an effective anti-VEGF monoclonal antibody (134). Moreover, single-agent treatment with bevacizumab contributed to striking tumor growth suppression of 20 diverse human tumor cell lines (13 different tumor types) implanted into nude mice regardless of the route of administration or the location of tumors (135). As such, it was believed that anti-angiogenesis therapeutic strategy was essential for repressing the growth of tumor and retarding metastasis by virtue of blocking the fuel supply and destroying the circulating principle routes for the tumor cells through prohibiting tumor angiogenesis. In spite of the promising preclinical data, the clinical outcomes of VEGF inhibition monotherapy for the treatment of solid tumors have been observed to be less than expected, with only limited objective response rates (ORRs) and a lack of meaningful survival benefits in phase 3 clinical trials (136). For example, in metastatic colorectal cancer, an ORR of 3.3% was calculated among chemotherapy-pretreated patients receiving bevacizumab, a well-known monoclonal antibody against human VEGF. On the contrary, a wide range of randomized phase 3 clinical trials of bevacizumab treatment in combination with systemic chemotherapy have shown profound enhancement in progression-free survival (PFS) and overall survival (OS) when in comparison to systemic chemotherapy alone (137–139). These clinical data seem to be counterintuitive. Anti-VEGF therapy is designed to induce the destruction of tumor blood vessels and thus tumor starvation, whereas the effectiveness of chemotherapeutic agents relies on adequate tumor blood flow and thereby efficient delivery of therapeutic drugs.

In 2005, “vascular normalization” theory was firstly proposed by Rakesh Jain, which potentially addresses this paradox (140). The theory postulates that instead of pruning blood vessels, the judicious employment of anti-angiogenic treatment reverses the structurally and functionally aberrant tumor blood vessels towards a more normal phenotype. The normalized tumor blood vessels tend to improve the treatment outcomes of chemotherapy, immunotherapy and radiotherapy since they offer reliable pathways for the anti-tumor elements penetrating into the tumor parenchyma efficiently (141, 142). Notably, the conception of vascular normalization possesses various limitations owing to the fact that it seems to be difficult to predict the accurate regimen (therapeutic window of dosage and time) that will confer vascular normalization rather than vessel pruning. In this regard, the same anti-angiogenic strategy (e. g. VEGF suppression), administrated at divergent doses or with the same dose in distinct tumor types, is able to harness vessel repression and further aggravate hypoxia or vascular normalization, and thereby reoxygenation in tumors (143, 144). For example, Huang and co-workers demonstrated that rational usage of anti-angiogenic treatment is crucial to ameliorate hypoxia in the tumor parenchyma and subsequently generate an immune-supportive TME (145). In their study, it was revealed that excessive treatment of anti-VEGFR2 tended to aggravate intratumoral hypoxia and restricted CD8^+^ T cell infiltration into the TME, thereby prohibiting anti-tumor immune response. Rivera and colleagues also unveiled that high-dose of anti-angiogenic treatment was able to result in the activation of PI3K signaling cascade in the myeloid cells that further attenuate immunity and bolster neovascularization (117). Hence, further preclinical and clinical studies are required to achieve the optimization of anti-angiogenic treatment in the era of cancer immunotherapy, aiming at opening the window of vascular normalization within the TME and fuel anti-cancer immune response.

### 4.2 Vascular normalization triggered by vasculature-targeting strategies potentiates cancer immunotherapy

Indeed, VEGF inhibition has been demonstrated to increase the number of tumor-infiltrating lymphocytes by virtue of resulting in tumor vascular normalization in both animal models and humans (146). In addition to repressing the classical VEGF-VEGFR signaling axis, targeting a plethora of alternative components to achieve tumor vascular normalization is also prone to exhibit striking therapeutic benefits. It has been increasingly recognized that fortifying vascular structure and enhancing vascular function can be fulfilled mainly through heightening pericyte coverage, restoring EC junctions, boosting tumor vascular perfusion and alleviating tumor hypoxia, thereby facilitating the infiltration of T cells and further provoking an immune-supportive TME. To this end, vascular normalization achieved by proper vasculature-targeting strategies has high propensity to improve cancer immunotherapy.

#### 4.2.1 Regulating pericyte coverage

**Figure 4:**
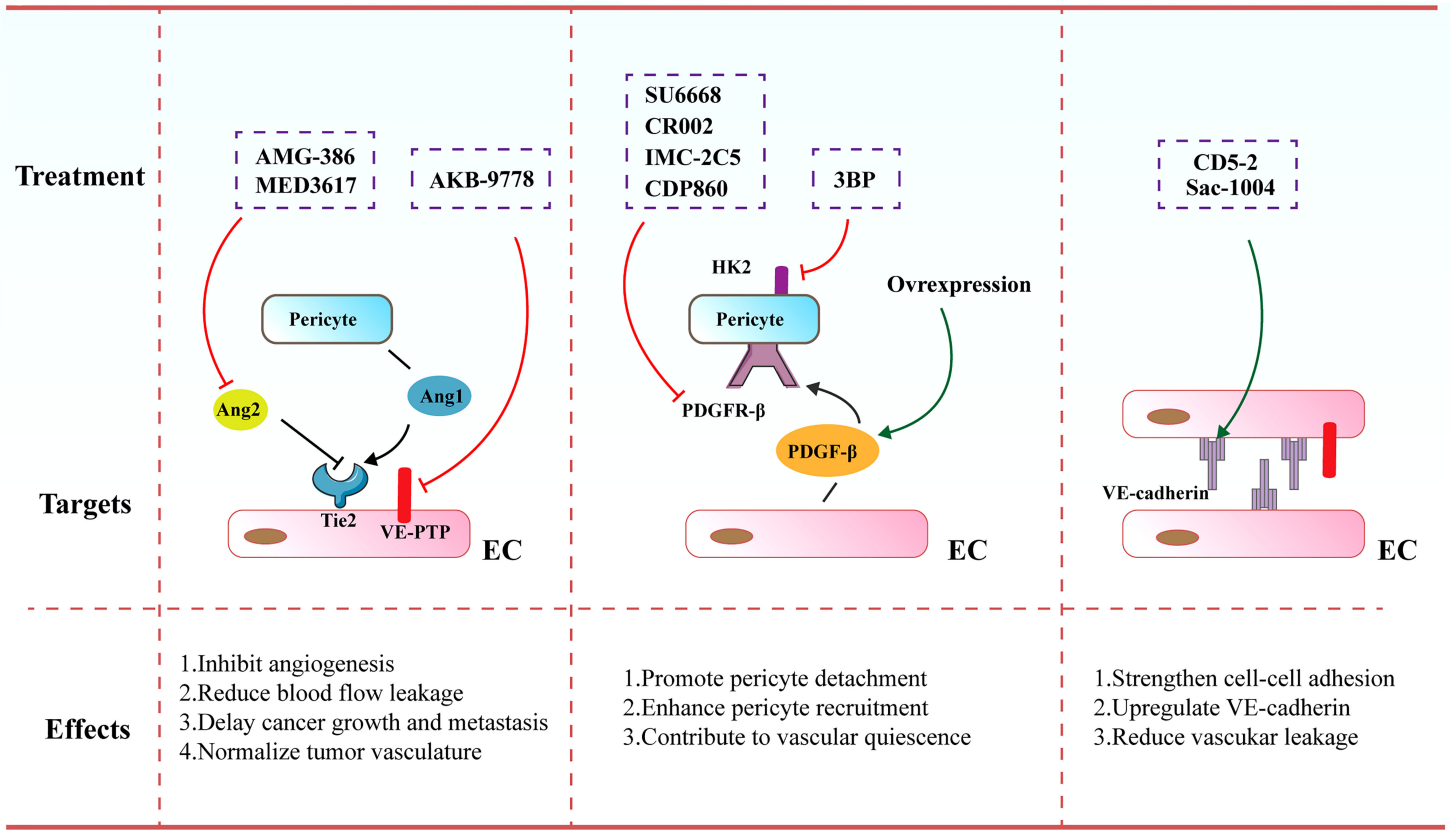
Therapeutic strategies and targets to normalize tumor blood vessels. Increased pericyte coverage, enhanced EC junctions, elevated tumor vascular perfusion as well as alleviated tumor hypoxia tend to result in the achievement of vascular normalization, further improving the distribution and efficiency of anti-cancer therapies.

Pericytes have been regarded as an indispensable element of normal blood vessels, in which they result in vascular maturation and integrity, but inadequate pericyte coverage is frequently observed in tumor vasculature. As such, orchestrating pericyte coverage has been perceived as an effective and efficient therapeutic strategy to normalize tumor blood vessels (147, 148). A list of critical signaling pathways that are exploited to modulate pericyte coverage have been outlined in **Figure 4**.

Angiopoietin (Ang) signaling cascade has been reported to participate in governing the vascular permeability, vasodilation and vasoconstriction. The angiopoietins and their receptor Tie-2 (TEK) exert vital effects on controlling blood vessel formation and remodeling. Ang-1 that is secreted by perivascular cells plays an important role in driving maturation of blood vessels. In contrast, Ang-2 mainly secreted by ECs is involved in the disruption of EC-pericyte interaction ECs as it harbors antagonistic function to the Tie-2 receptor. Various inhibitors targeting the Ang/Tie signaling cascades have been formulated and are undergoing investigations in preclinical and clinical studies. For instance, MEDI3617 as an Ang-2 antibody was shown to lead to the reduction in tumor angiogenesis, a striking enhancement of chemotherapy, as well as impaired metastasis in multiple preclinical models including colorectal, renal, hepatocellular, and ovarian carcinoma (149). AMG-386 (trebananib), a peptide-Fc fusion protein, was observed to diminish tumor angiogenesis and further retard tumor progression *via* interfering with the binding of Ang-1 and Ang-2 to the Tie-2 receptor (150). Furthermore, Koh and colleagues conducted a comparison of Ang-2-Binding and Tie-2-Activating Antibody (ABTAA) and Ang-2-Blocking Antibody (ABA) in mice implanted with various types of tumors. ABTAA favorably resulted in immune reprogramming in tumors, implying that simultaneous Tie-2 activation and Ang-2 suppression potently confer a favorable TME to facilitate the delivery of chemotherapeutic drugs into tumors (151). Furthermore, Jain and co-workers treated glioblastoma-bearing mice with antibodies targeting Ang-2/VEGF (CrossMab, A2V), and found that A2V attenuated microvascular density and polarized M2-like TAMs toward the anti-tumoral M1-like TAMs, leading to the retardation of tumor progression (152).

Of note, vascular endothelial protein tyrosine phosphatase (VE-PTP) as a specific EC-phosphatase tends to dephosphorylate and further inactivate Tie2. AKB-9778 emerges as a selective and potent VE-PTP inhibitor that triggers the activation of Tie2 in ECs. The treatment of AKB-9778 mitigated the growth of breast cancer and diminished the size, number and progression of metastatic foci (153). Further, Vestweber et al. demonstrated that silence of VE-PTP with AKB-9778 and genetic strategy declined vascular permeability mediated by inflammatory factors. Additionally, leukocyte transmigration across the endothelial barrier was prevented. Mechanistical analysis implicated that suppression of VE-PTP restored endothelial junctions through activating Tie-2, which yielded the activation of Rap1 and further conferred the dissolution of radial stress fibers *via* Rac1 and inhibition of nonmuscle myosin II (154).

PDGF/PDGFR signaling cascade are also reported to be involved in cancer progression *via* autocrine stimulation of cancer cell proliferation and paracrine effect on stromal cells triggering tumor angiogenesis. Notably, it was demonstrated that anti-angiogenic resistance was partially owing to robust mural cell proliferation and elevated PDGF-B level. SU6668 serves as a PDGFR-β inhibitor that eliminates tumor vasculature through propelling the detachment of pericytes from ECs. SU6668 in combination with SU5416 as a VEGFR inhibitor exhibited a synergistic effect on tumor eradication (155, 156). Decrease in intratumoral pressure and alleviated hypoxia ascribed to increased pericyte coverage are the driving forces for augmented T cell infiltration. Likewise, vascular normalization contributes to an elevated and uniform distribution of adhesion molecules on the luminal surface of ECs lining the tumor blood vessels, thereby facilitating more efficient docking and rolling of T cells, both of which promote the infiltration of T cells into tumor parenchyma (157). Of note, PDGFR-β inhibitors contained various antibodies, such as CR002, IMC-2C5 and CDP860. They are engineered Fab’ fragment–polyethylene glycol conjugates that target PDGFR-β and repress its activation (158, 159). However, a number of studies demonstrated that depletion of the pericytes-associated blood vessels is not more effective than anti-VEGF antibody alone (160). Further, loss of pericyte coverage hampered vessel maturation and promoted metastasis. As such, an alternative efficient therapy to induce vascular normalization seems to activate PDGF/PDGFR-β signaling cascade. Ellis and colleagues highlighted that overexpression of PDGF-B in mouse models of colorectal and pancreatic cancer resulted in the suppression of tumor growth through boosting the recruitment of pericytes and limiting the proliferation of ECs. Further, treatment with imatinib mesylate (PDGFR-β inhibitor) led to impaired pericyte coverage, which was associated with the destabilization of tumor blood vessels (161).

Moreover, Wong and co-workers elucidated that increased hexokinase 2 (HK2)-mediated pericyte glycolysis was capable of strengthening ROCK2-MLC2 axis-mediated contractility, thereby resulting in disrupted tumor vascular function (162). Treatment of a HK2 inhibitor (3BP) conferred the recruitment of pericytes, thereby facilitating tumor vasculature remodeling and further enhancing drug delivery into tumors (162). Additionally, genetic elimination of regulator of G-protein signaling 5 (RGS5) enabled immature PDGFR-β^+^ pericytes to turn into mature αSMA^+^NG2^+^ pericytes without influencing their overall coverage onto the blood vessels (163, 164). This phenotypic alteration in tumors eventually diminished hypoxia and vascular permeability, resulting in significant penetration of immune effector cells into the tumor parenchyma.

#### 4.2.2 Enhancement of endothelial junctions

It has been accepted that VE-cadherin acts as a central molecule for maintaining EC junctions and vascular integrity. Disruption of VE-cadherin expression or membrane location leads to augmented vascular permeability, reflecting its pivotal role in governing vascular barrier function (165, 166). Sac-1004 was a recently formulated molecule to build up appropriate IFP, ameliorate vascular permeability and enhance vascular perfusion. It intensified adherens junctions *via* modulating the cAMP/Rac/Cortactin signaling cascade to generate a cortical actin ring, which was able to boost the expression level of VE-cadherin (167). The dissociation of VE-cadherin from VE-PTP is a master regulatory factor of leukocyte migration across the endothelial barrier. The majority of leukocytes including lymphocytes transmigrate across the endothelium *via* a paracellular pathway, in comparison to the alternative transcellular route that occupies approximately 10% of transendothelial migration (168, 169). Vockel et al. demonstrated that upon α4β1 integrin in lymphocytes binding to endothelial VCAM-1, an important signaling pathway was initiated that resulted in the activation of Rac1. Rac1 subsequently triggered the activation of NOX, which transformed molecular oxygen to reactive oxygen species (ROS). These ROS activated the kinase Pyk2, which contributed to the phosphorylation of an undefined substrate for VE-PTP and further provoked VE-PTP to dissociate from VE-cadherin. This dissociation was correlated to elevated leukocyte transmigration and augmented vascular leakage (170).

Further, Zhao et al. uncovered an oligonucleotide-based inhibitor (171), which disrupted the interaction of microRNA-27α with its target VE-cadherin, leading to increased VE-cadherin expression (172, 173). Administration of CD5-2 in tumor-bearing mice boosted the expression of VE-cadherin in tumor endothelium, activating Tie-2 and tight junction pathways and normalizing vascular structure and function. CD5-2 treatment upregulated the levels of chemokines including CCL-2 and CXCL-10 that are involved in leukocyte transmigration, which facilitated the penetration of CD8^+^ T cells into tumor parenchyma (172). Qian et al. showed that Salvianolic acid B (SalB) elicited vascular normalization in the mouse models of breast cancer. More interestingly, SalB in combination with anti-PD-L1 inhibited the growth of tumors, which was mainly owing to increased infiltration of CD8^+^ T cells and enhanced delivery of anti-PD-L1 into tumors. Mechanistically, tumor cell enhancer of zeste homolog 2 (Ezh2)-driven cytokines perturbed the endothelial junctions with decreased VE-cadherin expression, which was reversed following the treatment of SalB (174).

#### 4.2.3 Modulation of vascular perfusion and hypoxia

Intriguingly, multiple studies showed that radiotherapy or chemotherapy could modulate the tumor vasculature and further promote vascular perfusion. Recently, it was revealed that fractionated radiation therapy profoundly augmented tumor vascular perfusion following two weeks of treatment. It was correlated to how quality of tissue viability and diminished hypoxia, but the increased VEGF level was counteracted in the presence of sunitinib (175). Gold nanoparticles (AuNPs) have been regarded as a promising nanomaterial resulting from their efficient drug-delivery properties and robust anti-tumor function. Huang et al. unveiled the anti-neoplastic activity of an improved targeting polymer and folic acid-modified gold nanoparticles (AuNPP-FA). AuNPP-FA was capable of normalizing tumor blood vessels *via* boosted vascular perfusion, alleviated hypoxia as well as improved EC junctions that was mediated by increased VE-cadherin expression in ECs. In this sense, the immunotherapeutic outcomes were significantly potentiated owing to the escalated penetration of CD8^+^ T cells. Mechanistic analysis revealed that AuNPP-FA reinforced the expression and production of semaphorin 3A (SEMA3A) in cancer cells, which further led to the suppression of Smad2/3 signaling cascade in ECs (176). Chloroquine (CQ), commonly employed as an autophagy inhibitor, was also observed to regulate tumor vasculature (177). CQ influenced the TME through promoting vascular perfusion, diminishing hypoxic area, and restraining the dissemination and intravasation of cancer cells. The vascular normalization effect of CQ predominantly relied on changes in the trafficking of endosomal Notch1 and its downstream signaling activation in ECs, which could be abrogated upon the silence of Notch1 in ECs (177).

Further, it has been held that the oxygen-sensing prolyl hydroxylase domain proteins (PHD1-3) are capable of targeting hypoxia-inducible transcription factors (HIFs) for degradation. Under the condition that oxygen tension drops, PHDs tend to be less active, in which case stabilized HIFs induce an adaptive response including angiogenesis. Nevertheless, aggravated hypoxia in tumors leads to excessive secretion of pro-angiogenic factors and subsequently tumor angiogenesis. Carmeliet et al. showed that haplodeficiency of PHD-2 failed to influence tumor vascular density and lumen size, whereas enhanced the endothelial junctions and vascular maturation. This contributed to improved tumor vascular perfusion and oxygenation, further inhibiting tumor cell invasion and metastasis. These normalized tumor blood vessels phenotypes relied on HIF-mediated upregulation of (soluble) VEGFR-1 and VE-cadherin (178). Moreover, Mazzone et al. illustrated that the B55α/PP2A complex limited PHD-2 function and enhanced the survival of ECs in a HIF-dependent manner. Furthermore, it tended to dephosphorylate p38 to protect ECs from the occurrence of cell stress including the onset of blood flow. Their data emphasize a specific role of the B55α/PP2A phosphatase complex in tumor vascular remodeling and suggested the promising application values of PP2A-inhibitors as robust anti-angiogenic agents exclusively targeting nascent blood vessels with a mode-of-action complementary to anti-VEGFR therapies (179).

### 4.3 Immune reprograming-mediated tumor vascular normalization

Intriguingly, despite that immune cells have been proposed to encrypt the functions of ECs, thereby having profound consequences on curbing tumor angiogenesis, it has been recently demonstrated that the activation of a series of certain types of immune cells could also exert essential effects on improving tumor vascular normalization (**Table 2**) (180, 181, 187).

**Table 2:** Immunotherapies inducing vascular normalization.

Immunotherapy	Drug treatment dosages	Tumor model	Functions	Refs
ICB	125 μg anti-CTLA-4, 125 μg anti-PD-1	Breast cancer	Activate Th1 cells to produce IFN-γ, increase the coverage of pericytes and normalize tumor blood vessels	(180)
ICB	5 mg/kg anti-CTLA-4, 10 mg/kg anti-PD-1, 250 μg/mouseanti-IFN-γ	Breast cancer colon cancer	Activate CD8^+^ T cells to produce IFN-γ, increase the coverage of pericytes and normalize tumor blood vessels	(181)
Anti-CTLA-4	5 mg/kg anti-CTLA-4	Breast cancer	Mediate vascular remodeling	(182)
Low-dose TNF-α	2 μg and 25 μg IFN-γ/IFN-γ-RGR, 0.2 ng, 0.2 μg and 2 μg TNF-α/TNF-α-RGR	Pancreatic neuroendocrine tumors	Stabilize the vascular network and improve vessel perfusion	(183)
Dual anti-PD-1/VEGFR-2 therapy	10 mg/kg anti-PD-1	Hepatocellular carcinoma	Promote normalized vessel formation	(184)
STING agonists (cGAMP or RR-CDA)	10 μg 3′3′- CGAMP, 25 μg RR-CDA	Breast and colorectal cancer	Up-regulate type I/II interferon genes and vascular stabilization genes	(185)
IFN-β	1.5×10^10^ vector particles of AAV2/8 CAG hIFN-β	Glioma	Improve intratumoral oxygenation and enhance the antitumor activity of ionizing radiation	(186)

It is noteworthy that, in addition to orchestrating immune-induced tumor cell elimination, ICB was also documented to provoke tumor vessel normalization at least in the orthotopic breast and ectopic colon cancer models. In both preclinical models, it was observed that repression of CTLA-4 or PD-1 resulted in diminished tumor vascular density, enhanced vascular perfusion, as well as alleviated hypoxia in the tumor bed, all of which are perceived as the central features of tumor vascular normalization (72, 180). In the both CD4^−/−^ and CD8^−/−^ mouse tumor models, it was shown that suppression of CTLA-4 and PD-1 was able to induce tumor vascular normalization through activating the Th1 cells. Nonetheless, it was deciphered that CD4^+^ T cells alone failed to sufficiently give rise to the remodeling of tumor blood vessels (180). Conversely, the deletion of CD4^+^ T cells contributed to the recruitment and accumulation of CD8^+^ T cells as well as elevated levels of IFN-γ, with a markedly tumor vascular normalization phenotype. In this regard, Zheng and co-workers drew the conclusion that the vascular normalization effect mediated by ICB therapy was potentially associated with the activation of CD8^+^ T cells induced by the IFN-γ signaling cascade (181). Additionally, Huang and colleagues highlighted that augmented release of Delta-like 1 (DLL1) in the TME gave rise to durable vascular normalization to diminish tumor hypoxia and facilitated the infiltration of CD8^+^ T cells as well as the polarization of M1-phenotypic TAMs. Mechanical analysis revealed that *in vivo* knockout of CD8^+^ T cells or silence of IFN-γ reversed the suppression of tumor growth and attenuated DLL1-mediated vascular normalization without influencing DLL1-induced macrophage polarization. Collectively, the data implicated that increased DLL1 levels in the TME conferred long-term tumor vascular normalization in a CD8^+^ T cell- and IFN-γ- dependent manner and enhanced the effectiveness of ICB (188).

Furthermore, Huang and co-workers also demonstrated that activated CD11b^+^Gr1^lo^F4/80^+^Siglec-F^+^ eosinophils were capable of yielding tumor vascular normalization, which further provide robust support to provoke tumor elimination by virtue of CD8^+^ T cells. Of note, the underlying mechanism that eosinophils triggered tumor vascular normalization remained elusive, whereas it might be due to that eosinophils skewed polarization of TAMs away from M2-like into M1-like profile through activating IFN-γ and TNF derived from eosinophils, leading to impaired secretion of VEGF. The normalized tumor vasculature enabled T cells to penetrate into tumors, which contributed to the formation of a positive feedback loop that promoted further polarization of TAMs to M1-phenotypic profile, as well as vascular normalization to facilitate more efficient T cell infiltration (182).

In addition to the above-mentioned immune-associated signaling cascades, there are also other elements that may normalize tumor vasculature. Recently, Yang and colleagues identified the stimulator of IFN gene (STING) agonists (cGAMP and RR-CDA) that activated the expression of STING in tumors to normalize tumor vasculature (185). Also, IFN-β was observed to be a potent anti-angiogenic cytokine that limited EC proliferation and subsequently triggered the maturation of tumor blood vessels *via* upregulating Angpt1 (37). Administration of intratumoral STING agonist contributed to the activation of type I IFN signaling together with the boosted expression levels of vascular integrity-related genes, such as Ang1, PDGFR-β and collagen-α. The transcriptional alterations ultimately resulted in tumor vascular normalization with increased pericyte coverage and more intact basement membrane support, thereby improving the penetration of CD8^+^ T cells into tumor sites and diminishing hypoxic area in the TME. Nonetheless, prohibition of the type I IFN signaling cascade *via* IFN-α/β receptor blockade almost entirely abrogated STING-mediated transcriptional alterations and also phenotypic changes in blood vessels. These data implicated that the elevated expression levels of vascular integrity-associated molecules in the STING-induced vascular remodeling partially relied on the function of type I IFN.

## 5 Anti-angiogenic therapy meets immunotherapy

### 5.1 ICB plus anti-angiogenic therapy in preclinical studies

It has been well accepted that, on one hand, tumor immune evasion is tightly associated with angiogenesis. On the other hand, tumor angiogenesis is thought to rely substantially on immunosuppressive tumor microenvironment (18, 189, 190). The potential to combine anti-angiogenic therapy with immunotherapy, in particular with immune checkpoint inhibitors, has been corroborated by numerous combinations in various preclinical mouse models (**Table 3**). However, apart from decreased IFP and correspondingly improved T cell infiltration into tumor parenchyma, we are not able to rule out other mechanisms by which ICB and anti-angiogenesis synergistically kill tumor cell. Thus, further explorations should be conducted in expanding models. To date, multiple mechanisms have been found to be related to synergistic effects.

**Table 3:** Preclinical studies testing combinations of anti-angiogenic agents and immunotherapies.

Immunotherapy	Antiangiogenic therapy	Tumor model	Key results	Refs
Anti-PD-1 mAb (clone RMPI-14; 10 mg/kg three times per week)	Vanucizumab	Breast cancer Melanoma Pancreatic cancer Neuroendocrine cancer	Decrease Tumor growth and increase animal survival	(191)
Anti-PD-L1 mAb (clone 10F.9G2;10 mg/kg thrice a week)	Anti-VEGFR2 mAb	Orthotopic HCC mouse	Reprogram the immune microenvironment and enhance vessel normalization	(184)
Anti-PD-1 mAb (clone RMPI-14; 10 mg/kg twice a week)	Anti-VEGFR2 mAb	MMTV-PyMT	A dose-dependent synergism	(192)
Anti-PD-1 mAb (clone RMPI-14; 0.25 mg every other day)	Anti-VEGFR2 mAb	Colon cancer	Inhibit Angiogenesis and enhance T cell infiltration	(193)
Anti-PD-1 mAb (clone RMP1-14; 0.25 mg twice a week)	Sunitinib	Colon cancer	Decrease PD-1^+^CD8^+^ T cells and enhance anticancer activity	(194)
Anti-PD-L1 mAb (clone 10F.9G2;10 mg/kg twice a week)	Anti-VEGFR2 mAb	Pancreatic cancer Breast cancer Glioblastoma	Increase IFN-γ-expressing CD8^+^ T cells and induce formation of HEVs *via* LTβR	(195)
Anti-PD-1 mAb (clone RMP1-14; 0.15 mg twice a week)	Anti-CD93 mAb	KPC tumors Melanoma	Decrease Tumor growth and increase T cell infiltration	(196)
Anti-PD-L1 mAb (10F.9G2; 5 mg/kg twice a week)	Sunitinib, Regorafenib, CVX-241, Anti-VEGFR2 mAb	Multiple orthotopic human tumor xenografts and syngeneic mouse tumor models	Decrease tumor growth and metastatic progression	(197)

#### 5.1.1 Inducing formation of high endothelial venules

Allen and colleagues explored the effects of anti-PD-L1 (B20S) therapy in combination with anti-VEGFR2 (DC101) in mice bearing mammary carcinoma, glioblastoma and pancreatic neuroendocrine tumor. It was observed that the combination therapy exhibited robust synergistic effects on tumor eradication and OS compared with monotherapy in pancreatic neuroendocrine tumor and mammary carcinoma (195). After the combination therapy of anti-PD-L1 and anti-VEGFR2 for two weeks, the expression levels of IFN-γ^+^CD8^+^ and IFN-γ^+^CD4^+^ T cells were dramatically boosted in the pancreatic neuroendocrine tumor and mammary carcinoma, whereas the number of IFN-γ^+^CD8^+^ T cells was only modestly elevated for approximately 50% in glioblastomas (195). As the direct barrier for the extravasation T cells, abnormal blood vessels in tumors are recognized as the determinant resulting in the restricted T cell infiltration in glioblastomas. In addition to augmented pericyte coverage, the endothelium following the combination therapy was thickened with flat ECs instead of plump ECs, showing the specific property of the presence of HEVs. Immunohistochemical examination verified the phenotypic changes in ECs. Indeed, it has been commonly believed that HEVs are associated with lymphocyte homing (198, 199). Similarly, it was shown that intratumoral HEVs propelled the infiltration of T cells into tumor parenchyma. LTβR signaling cascade was found to be critical to give rise to the HEV phenotype. Activation of LTβR signaling cascade with its agonist accompanying to combination therapy could strikingly retard the progression of glioblastoma, suggesting the pivotal role of HEVs in the combination therapy (195).

#### 5.1.2 Reciprocal regulation of VEGF/VEGFR2 signaling inhibition and ICB therapy

Voron et al. depicted that VEGF was able to augment the expression of PD-1 by leading to the activation of VEGFR2-PLCγ-calcineurin-NFAT signaling cascade (194). Consistently, anti-VEGF antibody profoundly down-regulated the expression of a bunch of preponderant immune checkpoint molecules (e. g. PD-1, CTLA-4, and TIM-3) on the CD8^+^ T cells in tumors (194). To this end, anti-PD-1 antibody in combination with anti-VEGF antibody was able to tremendously inhibit PD-1/PD-L1 signaling axis and synergistically delay the growth of tumor in particular for tumor with high VEGF levels in a synergistical way (194). Additionally, Ullrich and co-workers elucidated that, in a mouse model of small cell lung cancer (SCLC), anti-VEGF in combination with anti-PD-L1 synergistically enhanced the therapeutic outcomes in terms of the tumor progression compared with anti-PD-L1 or anti-VEGF monotherapy. Mechanistical studies revealed that VEGF-A was prone to elevate the co-expression of TIM-3 as an inhibitory receptor on T cells, suggesting the immunosuppressive role of VEGF in SCLC patients in the presence of anti-PD-1 therapy. This exhausted phenotype of T cells by PD-L1 blockade could be reversed following the supplement of anti-VEGF therapy (200).

#### 5.1.3 Overcoming the resistance and negative feedback of anti-angiogenesis and ICB

In addition to VEGF/VEGFR2 signaling cascade, Ang-2/Tie-2 is recognized as an alternative pro-angiogenic pathway that emerges as a substitute when encountering resistance to anti-VEGF therapy (201–203). Schmittnaegel et al. illustrated that the dual inhibition of VEGF and Ang-2 by a bispecific antibody A2V displayed a more robust therapeutic function compared to monotherapy (191). Upon the treatment of A2V, the numbers of tumor-killing immune cell subsets including mature DCs, M1-like TAMs and IFN-γ^+^/CD69^+^ CD8^+^ T cell were significantly enhanced (191). In the meanwhile, elevated number of perivascular CD8^+^ T cells was found to be accompanied to the high level of PD-L1 on the tumor cells, which was responsible for the IFN-γ-induced negative feedback modulating principle (191). Accordingly, the combination therapy of anti-PD-1 and A2V destructed the negative feedback loop and amplified the immune response in tumors (191). Moreover, anti-angiogenic treatment was also capable of ameliorating resistance to anti-PD-1 therapy through precluding the TOX-induced T-cell exhaustion phenotype in the TME. Kim and colleagues elucidated that VEGF could conspicuously increase the expression of the transcription factor TOX, which further governed the characteristics and functions of CTLs (204). It was shown that the TOX-induced transcriptional event led to compelling T-cell exhaustion and boosted the expression levels of a series of immune checkpoints including PD-1, TIM-3, LAG-3, and TIGIT, thereby attenuating the secretion of killing cytokines from CTLs (204). Intriguingly, anti-VEGFR2 in combination with anti-PD-1 exerted striking effect on elevating the immunotherapeutic effectiveness and T-cell revitalization. Taken together, combination therapy of anti-angiogenic drugs and ICB has emerged as a promising therapeutic strategy in the anti-PD-1-resistant cancer.

### 5.2 ICB plus anti-angiogenic therapy in clinical studies

As discussed above, the interaction between immune suppression and angiogenesis renders tumor immune escape and treatment resistance. As summarized above, on the basis of the promising data of preclinical studies, considerable clinical studies have been performed to determine the synergistic effect of ICB and anti-angiogenic therapy in cancer patients (**Table 4**). The details of **Table 4** can be obtained from http://clinicaltrials.gov/.

**Table 4:** Clinical trials examining the effects of ICB plus anti-angiogenic therapy.

Trials identifier	Disease	Treatment	Phase	Status
NCT03363867	OC	Atezolizumab + Bevacizumab + Cobimetinib	II	Recruiting
NCT02715531	Solid Tumor	Atezolizumab + Bevacizumab	I	Completed
NCT02366143	NSCLC	Atezolizumab + Carboplatin + Paclitaxel + Bevacizumab, Carboplatin + Paclitaxel + Bevacizumab	III	Completed
NCT02982694	MSI/CRC/APC/CR	Atezolizumab + Bevacizumab	II	Recruiting
NCT03074513	Several solid tumors	Atezolizumab + Bevacizumab	II	Active, not recruiting
NCT02921269	Cervical cancer	Atezolizumab + Bevacizumab	II	Completed
NCT03434379	Carcinoma/Hepatocellular	Atezolizumab + Bevacizumab, Sorafenib	III	Active, not recruiting
NCT02997228	CRC	Atezolizumab + Bevacizumab	III	Recruiting
NCT03024437	RCC	Atezolizumab + Bevacizumab + Entinostat	I/II	Active, not recruiting
NCT02724878	NCCKC	Atezolizumab + Bevacizumab	II	Active, not recruiting
NCT02839707	OC/FTC/PC	Atezolizumab + Bevacizumab + PLDH	II/III	Recruiting
NCT02659384	ON	Bevacizumab + Atezolizumab + Acetylsalicylic acid	II	Active, not recruiting
NCT02873962	PC/OC/FTC	Bevacizumab + Nivolumab + Rucaparib, Bevacizumab + Nivolumab	II	Recruiting
NCT02017717	RG	Nivolumab + Bevacizumab, Nivolumab + Ipilimumab	III	Active, not recruiting
NCT03386929	NSCLC	Avelumab + Axitinib + Palbociclib	I/II	Active, not recruiting
NCT02734004	OC/BC/SCLC/GC	MEDI4736 + Olaparib, Bevacizumab	I/II	Active, not recruiting
NCT03517449	EC	Pembrolizumab + lenvatinib	III	Active, not recruiting
NCT00790010	Melanoma	Ipilimumab + Bevacizumab	I	Active, not recruiting
NCT01950390	Melanoma	Ipilimumab + Bevacizumab	II	Active, not recruiting
NCT03671265	ESCC	SHR-1210 + Apatinib + Radiation	NA	Recruiting
NCT03502746	Mesothelioma	Nivolumab + Ramucirumab	II	Recruiting
NCT03606174	UC	Nivolumab + Sitravatinib	II	Recruiting
NCT02853331	RCC	Pembrolizumab + Axitinib	III	Active, not recruiting
NCT03680521	RCC	Nivolumab + Sitravatinib	II	Active, not recruiting
NCT03472560	NSCLC/UC	Avelumab + Axitinib	II	Active, not recruiting
NCT02493751	RCC	Avelumab + Axitinib	I	Completed
NCT02684006	RCC	Avelumab + Axitinib	III	Active, not recruiting

OC ovarian cancer, CR Chemotherapy Resistance, BC breast cancer, EC endometrial cancer, ESCC esophageal squamous cell carcinoma, FTC fallopian tube cancer, GC gastric cancer, NA not applicable, NCCKC Non-clear cell kidney cancer, NSCLC non-small cell lung cancer, PC peritoneal cancer, RCC renal cell cancer, SCLC small cell lung cancer, UC urothelial cancer, ON Ovarian Neoplasms, RG Recurrent Glioblastoma, CRC ColoRectal Cancer.

#### 5.2.1 Combination therapy of atezolizumab and bevacizumab

NCT02921269 is a phase II clinical trial for the combination therapy of atezolizumab (MPDL3280A) and bevacizumab in treating patients with recurrent, persistent or metastatic cervical cancer (205). Patients with advanced cervical cancer were intravenously injected with 15 mg/kg of bevacizumab and 1200 mg of atezolizumab every 3 weeks. In the entire evaluable patients (n=10), the disease control rate (DCR) by response evaluation criteria in solid tumors (RECIST) V.1.1 was 60% (0% complete response, 0% partial response, 60% stable disease). Median PFS was 2.9 months and median OS was 8.9 months (205). The combination of bevacizumab and atezolizumab did not meet the predefined efficacy endpoint, as addition of bevacizumab to PD-L1 blockade did not appear to enhance the ORR in cervical cancer.

However, The combination of atezolizumab and bevacizumab (NCT02715531) showed encouraging antitumor activity and safety in a phase 1b trial involving patients with unresectable hepatocellular carcinoma (206). Inspired by the encouraging outcome of NCT02715531, NCT03434379 is a phase III, open-label, randomized study of atezolizumab in combination with bevacizumab compared with sorafenib inpatients with untreated locally advanced or metastatic hepatocellular carcinoma (207). OS at 12 months was 67.2% (95% CI, 61.3 to 73.1) with atezolizumab-bevacizumab and 54.6% (95% CI, 45.2 to 64.0) with sorafenib. Median PFS was 6.8 months (95% CI, 5.7 to 8.3) and 4.3 months (95% CI, 4.0 to 5.6) in the respective groups (hazard ratio for disease progression or death, 0.59; 95% CI, 0.47 to 0.76; P<0.001). In short, atezolizumab combined with bevacizumab resulted in better overall and progression-free survival outcomes than sorafenib.

#### 5.2.2 Combination therapy of avelumab and axitinib

In 2018, Choueiri et al. firstly demonstrated the efficacy of avelumab plus axitinib (VEGFR1-3 TKI) in the treatment of RCC. NCT02493751 is a phase Ib study with the purpose to assess the safety, pharmacokinetics, and pharmacodynamics of avelumab (anti-PD-L1) plus axitinib therapy (208). For a total of 55 patients recruited in the study, 54 patients received avelumab plus axitinib therapy except for one patient due to abnormally increased blood creatine phosphokinase (208). Within a follow-up period of appropriate one year, 58% (32 of 55) patients displayed complete response or partial response to the combination therapy while 20% (11 of 55) patients had stable disease (208). Notably, it was found that PD-L1 level fail to dramatically influence the treatment effectiveness. No matter choosing cut-off value as 1% or 5%, the ORRs of PD-L1 high expression group and PD-L1 low expression group were comparable (208).

Inspired by the encouraging outcome of NCT02493751, a phase III clinical trial (NCT02684006) was carried out to compare the efficacy of avelumab plus axitinib vs. sunitinib (a multitargeted tyrosine kinase inhibitor) monotherapy in the advanced RCC (209–212). Treatment-naive patients with RCC were divided (1:1) to receive avelumab (10 mg/kg) intravenously every 2 weeks plus axitinib (5 mg) orally twice daily or sunitinib (50 mg) orally once daily for 4 weeks (6-week cycle). Of the 886 patients, 442 were randomized to the avelumab plus axitinib treated group and 444 to the sunitinib treated group. After a minimum follow-up of 13 months (data cut-off 28 January 2019), the PFS was significantly longer in the avelumab plus axitinib treated group than in the sunitinib treated group, but the OS data were still immature (211).

#### 5.2.3 Combination therapy alleviates adverse effects

ICB was shown to provoke the appearance of edema in patients with brain tumors, sometimes even leading to death (213). This phenomenon was different from the vessel normalization triggered by ICB therapy in various breast cancer models (180), which indicates that the vasculature-associated effects rely on the location and/or type of tumors. The possibility requires to be further explored. However, anti-VEGF drugs were able to alleviate the glioblastoma-related brain edema in both mice and patients (214, 215), offering a strong rationale for combining low-dose anti-VEGF therapy with immunotherapy in particular ICB therapy for potentially reducing the brain metastasis as well as ameliorating the side effects.

Of note, it has been observed that most adverse events of ICB therapy are reported to be closely associated with the hyperactive immune response, thereby displaying T cell mediated auto-immune like inflammatory disease (216, 217). Perturbed immune homeostasis by ICB therapy contributes to immune-mediated damage in normal tissues, including hepatic, gastrointestinal, and skin system (216). In general, it was demonstrated that the risk of adverse events of monoclonal antibodies that target either PD-1 or PD-L1 was lower than that of anti-CTLA-4 monoclonal antibody (grade 3-4 adverse event: 7-12% vs. 10-18%) (216). Interestingly, these adverse events could be mitigated through terminating the ICB therapy or diminishing the dosage of ICB (218). Theoretically, anti-angiogenic therapy leads to tumor vascular normalization, which generates a favorable microenvironment for T cell infiltration and drug delivery into tumors. However, in terms of the combination therapy of anti-angiogenesis and ICB, it was speculated that the positive feedback loop between the two therapies enabled reduced dose usage of ICB, which was sufficient to counteract immunosuppressive microenvironment with less adverse events (218).

## 6 Discussion

To date, it has been widely held that anti-angiogenic therapy is commonly used for the treatment of cancer. Nevertheless, various limitations including off-target effects, high effective doses and drug resistance have built up hurdles for the usage of anti-angiogenic therapies in patients with cancer. As such, novel vasculature-targeting therapeutic strategies with improved efficacy and safety are urgently required for preventing cancer progression in clinic (219, 220). With the burgeoning advancements in nanotechnology, smart nanotherapeutics have provided preponderant possibility to exclusively target tumor blood vessels. For instance, through specifically delivering thrombin loaded DNA nanorobots (Nanorobot-Th) into tumor blood vessels, an intratumoral thrombosis was initiated to trigger vascular infarction, and eventually tumor necrosis (221). Furthermore, anti-angiogenic drug resistance has been observed in clinic partially owing to the heterogeneity of tumor blood vessels. Tumor endothelial cells (TECs) are likely to be influenced by their parental tumors through the secreted tumor-derived factors, such as growth factors, chemokines, and microRNAs (222). TECs possibly differ on the basis of their TME, as well as tumor progression stage. To this end, it has the potential to develop the markers of TECs into biomarkers that characterize the heterogeneous tumor blood vessels, enabling the more rational selection of anti-angiogenic strategies. Multiple peptides that recognize TEC plasma membrane molecules have been identified. An asparagine-glycine-arginine (NGR) motif peptide ligand for CD13 has been shown to be overexpressed in TECs. NGR motif peptides have been successfully used to specifically deliver drugs as well as liposomes to the tumor blood vessels (223). Another peptide motif, named arginine-glycine-aspartic acid (RGD), has also been employed for drug delivery due to that it can recognize integrin αVβ3 that is highly expressed in TECs (224). Therefore, novel drug delivery systems to exclusively target heterogeneous tumor blood vessels have the potential to become promising anti-angiogenic strategies for avoiding side-effects and improving overall anti-cancer efficacy. Further in-depth studies on TEC heterogeneity may pave the way for facilitating the development of appropriate anti-angiogenic drug delivery systems.

It has been widely held that ICB has deemed to be a revolutionary milestone in light of cancer therapy. Nevertheless, a large number of cancer patients fail to respond to the ICB monotherapy, or even undergo relapse, sometimes harbor nonnegligible long-term toxicity, all of which have given rise to continuous attempts to address these issues by virtue of combination treatment of anti-angiogenesis and immunotherapy. As what we have outlined in this review, the interactions between tumor angiogenesis and immune suppression is very dynamic and a two-way process, facilitating the formation of positive feedback loop to propel the two processes reciprocally. To this end, modulation of either side is able to disrupt the vicious circle in the TME, further retarding the tumor progression. More specifically, Anti-angiogenic therapies in particular vascular normalization strategy are able to result in striking alterations in the tumor blood vessels of the TME, which tend to further potentiate the penetration and function of immune effector cells in tumors to fulfill efficient cancer immunotherapies. Moreover, the striking effects of ICB on the TME, in particular the tumor vascular alterations it triggers, have preference to offer new and realistic variables by which to examine and forecast the responses of tumor immunotherapy (225). There have been multiple lines of evidence verifying the effective and efficient clinical outcomes of the combination therapy of anti-angiogenesis treatment and ICB. Such promising data are required to be further confirmed in an array of currently ongoing randomized clinical studies with longer follow-up.

Of note, a bunch of points remain to be optimized aiming at orchestrating the effectiveness of combination therapy. Firstly, predictive clinical biomarkers are supposed to be discovered to lock in the subpopulation of cancer patients who are able to respond to the combination therapy. It is imperative to notice that the majority of the promising outcomes from the combination therapy of ICB and anti-angiogenic treatment have been predominantly observed in RCC, which is regarded as a high angiogenic and immunogenic tumor type with massive infiltrating immune cells (226). Secondly, it is also worthy of note that the core of anti-VEGF(R) agents as the predominant anti-angiogenesis strategies is needed to be diversified. The less expected clinical benefits propose the issue of resistance to anti-VEGF(R) therapy, which drives the proceedings in the strategies for modulating numerous pro-angiogenic signaling molecules, such as VEGFA-D, PDGF, and FGF. Thirdly, whether the functions of combination therapy of anti-angiogenic treatment and ICB are additive or synergistic is required to be investigated. Additionally, since the angiogenic profiles are distinctive depending on the types of organ, more solid evidence at the organ level should be obtained to increase the understanding on the responses to ICB. More importantly, a critical issue requires to be solved is the optimization of the dose and application arrangement of anti-angiogenesis during the combination therapy procedure. In fact, prolonging the therapeutic window of vascular normalization and controlling excessive vascular disruption tend to fulfill the maximal therapeutic benefit. Collectively, we believe that anti-angiogenesis treatment in combination with ICB is capable of working as an effective and efficient approach to brake the anti-cancer drug resistance, as well as increase the therapeutic outcomes and improve the prognosis of cancer patients.

## Author contributions

YaZ, YL and AW: Conceptualization and design the review, and review final version approval. WZ and CQ: Bibliographic research. WC, WZ, SY, and PS: Writing - original draft preparation. YT, YuZ and CY: Table and figure design. ZW, AW and YaZ: Supervision. YaZ, AW and SY: Funding acquisition. All authors contributed to the article and approved the submitted version.

## Funding

This research was funded by National Natural Science Foundation of China (Grant No.: 82003991, 81973587 and 81973734), Jiangsu Province Traditional Chinese Medicine Leading Talents Program (Grant No.: SLJ0229), Natural Science Foundation of Higher School of Jiangsu Province (Grant No.: 18KJA360007), National TCM Innovation Backbone Training Program (Chinese medicine practitioners letter (2019) No.128), the Open Project Program of Jiangsu Key Laboratory for Pharmacology and Safety Evaluation of Chinese Materia Medica (Grant No.: JKLPSE201812), and the Postgraduate Research & Practice Innovation Program of Jiangsu Province (SJCX22_0791 and KYCX21_1743).

## Conflict of interest

The authors declare that the research was conducted in the absence of any commercial or financial relationships that could be construed as a potential conflict of interest.

## Publisher’s note

All claims expressed in this article are solely those of the authors and do not necessarily represent those of their affiliated organizations, or those of the publisher, the editors and the reviewers. Any product that may be evaluated in this article, or claim that may be made by its manufacturer, is not guaranteed or endorsed by the publisher.
